# Evaluation of Survival and Post-operative Radiation Among Patients with Advanced Medullary Thyroid Carcinoma: An Analysis of the National Cancer Database

**DOI:** 10.1245/s10434-021-11158-9

**Published:** 2022-01-23

**Authors:** Thomas J. Ow, Vikas Mehta, Seokhwa Kim, Mayand Vakil, Patricia Friedmann, Haejin In

**Affiliations:** 1 Department of Otorhinolaryngology – Head and Neck Surgery, Montefiore Medical Center/Albert Einstein College of Medicine, Bronx, NY 10467; 2 Department of Pathology, Montefiore Medical Center/Albert Einstein College of Medicine, Bronx, NY 10467; 3 Gangdong Hana ENT Clinic, Hana ENT Hospital, Seoul, South Korea; 4 Department of Otolaryngology-Head and Neck Surgery, Rutgers New Jersey Medical School, Newark, NJ; 5 Department of Surgery, Montefiore Medical Center/Albert Einstein College of Medicine, Bronx, NY, 10467; 6 Department of Cardiothoracic and Vascular Surgery, Montefiore Medical Center/Albert Einstein College of Medicine, Bronx, NY, 10467; 7 Department of Epidemiology and Population Health, Albert Einstein College of Medicine, Bronx, NY, 10467

## Abstract

**Background:**

This study compares survival between patients with medullary thyroid cancer (MTC) treated with surgery (S) versus surgery and radiation (SRT).

**Methods:**

Patients from The National Cancer Database (NCDB) diagnosed between 2008-2016 with stage III/IV MTC, lymph node disease, and no distant metastases were studied. Kaplan-Meier analyses and log rank statistics were used to estimate and compare overall survival between patients treated with S versus SRT. Mutlivariable Cox proportional hazards models and propensity matching were used to adjust for confounding and selection bias.

**Results:**

Among 1370 patients, 1112 (81%) received S while 258 (19%) received SRT. The HR for mortality in the SRT group was 1.784 (95% CI 1.313, 2.43) after multivariable adjustment for confounding variables. Furthermore, SRT remained associated with higher mortality (p < 0.008) after propensity matching in an effort to adjust for selection bias.

**Conclusions:**

Analysis of NCDB patients showed that SRT is associated with significantly higher mortality among patients treated for stage III and IV MTC with positive lymph node disease. Though this observation can be attributed to unmeasured confounders or selection bias, the cause for the profound survival differences deserves prospective evaluation, especially as adjuvant therapies for this disease continue to evolve.

## Introduction

Medullary thyroid cancer (MTC) is rare, accounting for a minority (1-2%, 1000 cases per year) of all thyroid cancer cases diagnosed annually in the United States (US).^1, 2^ Approximately 80% of cases occur sporadically, with the remainder occurring in the context of inherited syndromes, namely Multiple Endocrine Neoplasia (MEN) Type 2A or Type 2B.^3^ While survival outcomes for patients that present with stage I – III disease range between 89%-98%, only 68% of those with stage IV disease survive 5 years.^4^ MTC that exhibits extrathyroidal extension, extensive lymph node involvement, and larger primary tumors are all associated with increased risk of locoregional recurrence, distant metastases and decreased survival.^5^

Due to MTC originating from the C-cells, adjuvant radioactive iodine is not an option in this disease. The mainstay of treatment remains surgical resection with or without lymphadenectomy, and the addition of adjuvant external beam radiation therapy (EBRT) to potentially improve outcomes for high-risk patients. Both the National Comprehensive Cancer Network (NCCN) and the American Thyroid Association (ATA) guidelines for medullary thyroid carcinoma management^2, 6^ indicate that external beam radiation therapy should be considered when there is grossly unresectable or microscopic residual disease, or for those patients deemed to be high-risk (i.e. extrathyroidal extension or extensive nodal involvement). However, the ATA guidelines delineate that this is a grade C recommendation (based on expert opinion) and the NCCN guidelines state that “adjuvant EBRT/IMRT is rarely recommended”. Improved locoregional control has been documented in retrospective data,^7–9^ but the benefit for survival remains largely unclear without any prospective trial data.

The largest reported MTC patient cohort assessing the effect of radiation therapy on survival in the adjuvant setting came from the Surveillance, Epidemiology and End Results Database.^10^ This study looked at survival outcomes in 534 patients from 1988-2004 and showed no improvement in overall survival with the addition of EBRT in all patients, and after multivariable adjustment carried out to examine the subset of patients with node-positive disease. Due to the improvement in radiation delivery techniques (i.e. intensity-modulated radiation therapy) and the collection of data from a larger percentage of the US population, we sought to perform a more recent analysis using the National Cancer Database (NCDB) to further assess the association of adjuvant radiation therapy with survival among patients with advanced MTC.

## Methods

### Patient Cohort

Data were acquired from patients registered within the NCDB, which contains information describing 29 million total cancer cases from 1500 Commission on Cancer (CoC)-accredited institutions across the US. The project was deemed exempt from institutional review board (IRB) review and approval since the study used deidentified information from registry data. The cohort was limited to patients with medullary thyroid cancer who were diagnosed with American Joint Commission on Cancer (AJCC) stage III and IV disease, who were free of distant metastases at diagnosis, and who had documented positive lymph node disease. The following specific inclusion and exclusion criteria were used to assemble the cohort:

### Inclusion Criteria

Data included patients ages 18 – 90 years old who were diagnosed with medullary thyroid carcinoma between 2008 – 2017, selected *using International Classifications for Diseases for Oncology*, Third Edition, codes “8345” *Medullary Carcinoma With Amyloid Stroma*, and “8510” *Medullary Adenocarcinoma*. Ultimately, patients diagnosed in year 2017 were excluded because of lack of follow up data in NCDB records at the time of analysis.

### Exclusion Criteria

Patients were excluded if the initial diagnosis was not made at the reporting facility, if distant metastasis was present at diagnosis (excluded patients with M1 disease), if lymph node metastases were not present at diagnosis/identified on pathology at the time of surgery (excluded patients with N0 disease), if patients were not staged as AJCC stage III or IV, and if no data were available for radiation treatment. For patients who received radiation treatment, patients who received radiation before surgery were excluded. Additionally, if patients received radiation greater than 180 days after surgery, they were excluded in order to limit inclusion of patients who may have received radiation for a later local or regional recurrence.

### Data Collection

The following variables were extracted and collected for analysis: age at diagnosis, gender, race and ethnicity (categorized as non-Hispanic White, non-Hispanic Black, Hispanic, and Other), Charlson-Deyo Score (0, 1, 2 or 3), insurance status (none, private, Medicaid, Medicare), estimated income level (<$30,000, $30,000 - $36,000, $36,000 - $46,000, >$46,000, and unknown – NCDB extrapolates this variable using median household income for patient’s zip code based on census 2000 data), year of diagnosis (categorized [2008 – 2011], [2011 – 2014], [2014 – 2017]), surgical margin status (negative vs. positive), extrathyroidal extension (negative vs. positive), number of lymph nodes positive, tumor grade, lymphovascular invasion, AJCC T-stage, AJCC N-stage, AJCC M-stage, and AJCC overall stage group, vital status, and time from diagnosis to time of death or time of last contact. For sub-cohort analyses, the study cohort was stratified based on the number lymph nodes positive categorized by quartiles (1-3 nodes, 4-7 nodes, 8-16 nodes, >16 nodes).

### Statistical Analyses

Categorical variables were summarized using counts and percentages. Univariate associations comparing S to SRT groups were assessed using Chi-Square tests. Survival curves were estimated using the Kaplan-Meier method, and statistical significance between strata were determined using log rank test.

We used two different analytic methods to conduct a robust comparison of survival between the two treatment groups. Multivariable Cox proportional hazards regression was used in the original cohort to estimate the hazard ratio (HR) for mortality associated with treatment (S versus SRT) after adjusting for confounding variables. If variables were highly correlated with other covariates in the model these were adjudicated and one of the correlated variables was selected to remain in the model.

Additionally, we used propensity score matching to balance the S and SRT groups on the following known and available covariates: year of diagnosis (grouped into 3 equal periods), gender, race, Charlson-Deyo Category, income category, type of insurance, AJCC T-stage, number of positive lymph nodes (grouped into quartiles, as above), and surgical margin status. Propensity score matching was carried out using the “greedy” method with a caliper of 0.10. Kaplan-Meier analyses and cox proportional hazards models for overall survival were also then carried out on the propensity-matched cohort (in addition to the original cohort, as described above). For all analyses, a p-value <0.05 was accepted as the threshold for statistical significance. All statistical analyses were carried out using SAS 9.4 software (SAS Inc, Cary, NC).

## Results

**Figure 1.**
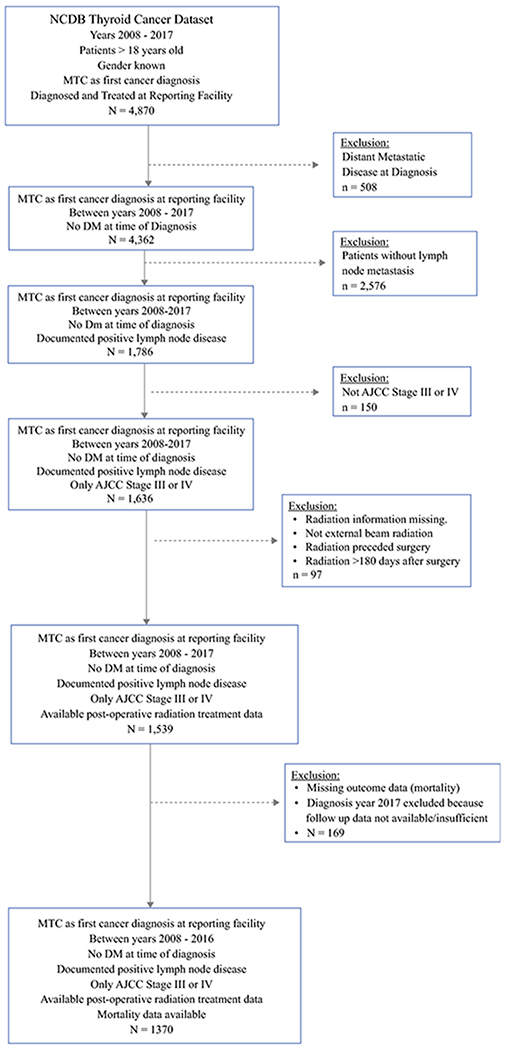
Consort diagram describing exclusions used to develop the study cohort composed of patients reported in the National Cancer Database (NCDB) with medullary thyroid cancer (MTC) diagnosed between 2008 – 2016 with lymph node – positive, American Joint Commission on Cancer (AJCC) Stage III or IV disease, with no distant metastasis (DM) at the time of diagnosis, and data available confirming adjuvant radiation or no radiation was delivered.

The inclusion and exclusion criteria used to generate the cohort, with the specific numbers of patients excluded at each step, are presented in Figure 1. The cohort studied consisted of 1370 patients with medullary thyroid carcinoma diagnosed and treated at a NCDB site between 2008 - 2016, who had lymph node metastases and no distant metastasis at the time of diagnosis. Among these patients, 1112 (81%) received surgery alone for treatment, while 258 (19%) received surgery followed by radiation. Older age and male gender were significantly associated with radiation treatment rendered, and as would be expected, receipt of radiation was significantly associated with positive surgical margins, extra-thyroidal extension, higher T-stages, greater number of lymph nodes positive, and AJCC stage IV disease. Variables for tumor grade and lymphovascular invasion had missing data for large numbers of patients and thus were not included in the subsequent analyses. The patient, treatment and pathologic characteristics of the study cohort, along with the association of these variables with S versus SRT, are presented in Table 1.

**Figure 2.**
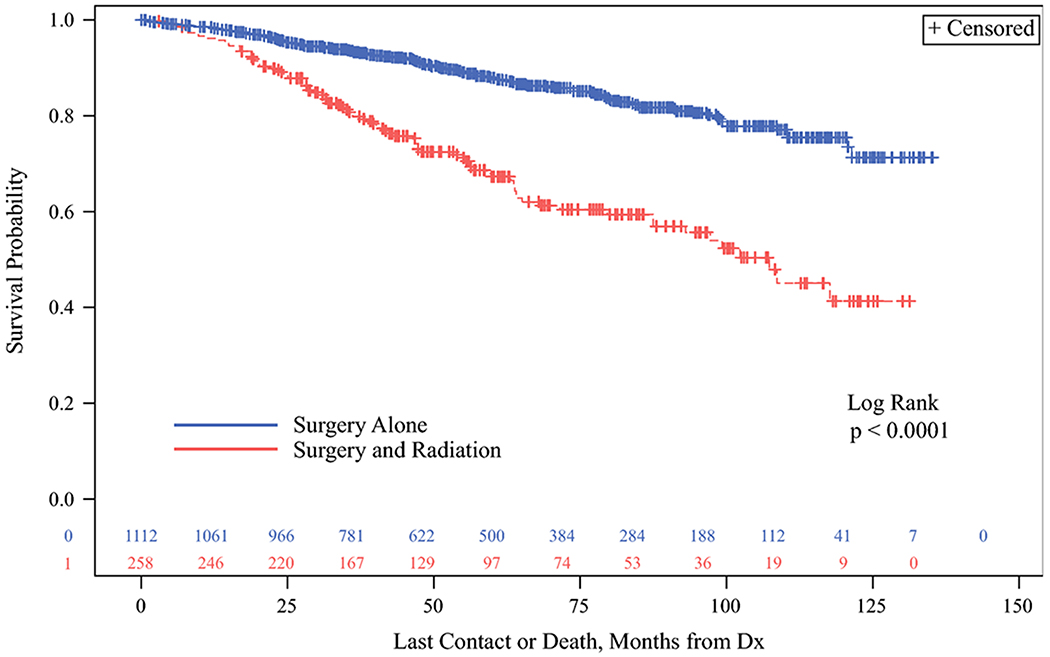
Kaplan-Meier analysis showing overall survival probability comparing patients with medullary thyroid cancer in the NCDB cohort who underwent surgery compared to those that underwent surgery and radiation. The surgery and radiation group demonstrated significantly decreased overall survival (p<0.0001). Abbreviation: Dx – diagnosis.

Overall survival was evaluated for the study cohort. The probability of overall survival was significantly lower among those that received SRT compared to those that received S, (Figure 2, Log Rank p <0.0001). The association of SRT with worse survival remained significant after adjusting for competing factors in multivariable Cox proportional hazards regression analysis, with a HR 1.784 (95% CI 1.313, 2.426, p=0.0002) (Table 2).

### Propensity Matched Analysis

**Figure 3.**
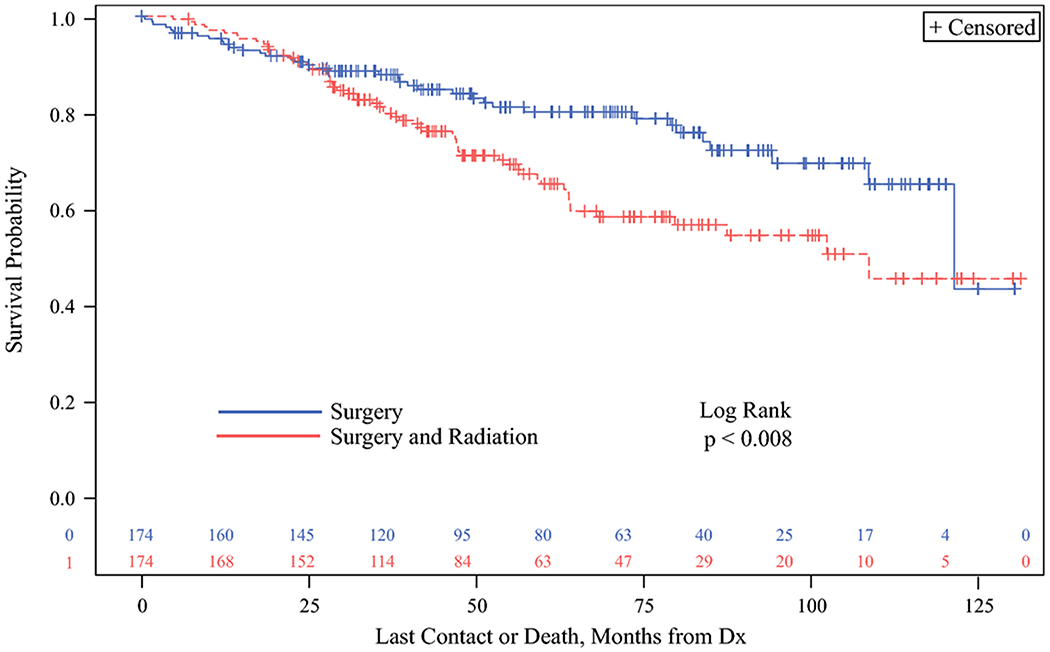
Kaplan-Meier analysis showing overall survival probability comparing patients with medullary thyroid cancer in the NCDB cohort who underwent surgery compared to those that underwent surgery and radiation among the propensity score matched cohort. The surgery and radiation group demonstrated significantly decreased overall survival (p<0.008). Abbreviation: Dx – diagnosis.

To adjust for selection bias, propensity matching was used to balance the two treatment cohorts using the following covariates: year of diagnosis (grouped into 3 equal periods), gender, race, Charlson-Deyo Category, income category, type of insurance, AJCC T-stage, number of positive lymph nodes (grouped into quartiles, as above), and surgical margin status. Propensity score matching resulted in 174 patients who received S were matched to 174 patients who received SRT. In the matched cohort, SRT remained associated with decreased overall survival on Kaplan-Meier analysis (Log Rank p = 0.008, Figure 3). Regression using Cox proportional hazards modeling showed that postoperative radiation was associated with worse overall survival with a HR of 1.738 (95% C.I. 1.152, 2.621). As can be seen on the Kaplan-Meier analysis (Figure 3) survival probability between the S and SRT groups appeared to cross and diverge after two years from treatment, suggesting that the hazards were not constant over time. Therefore, we also examined the HR associated with SRT compared to S for discrete time points based on the observed KM estimates of mortality. The HR for SRT at 1, 2, 3, 4, 5 and 6 years from treatment was 0.62, 0.98, 1.55, 1.80 and 1.75 respectively compared to S at these timepoints, indicating that survival may be better in years 1 & 2, but worse survival was consistently observed in years 3 and beyond.

**Figure 4.**
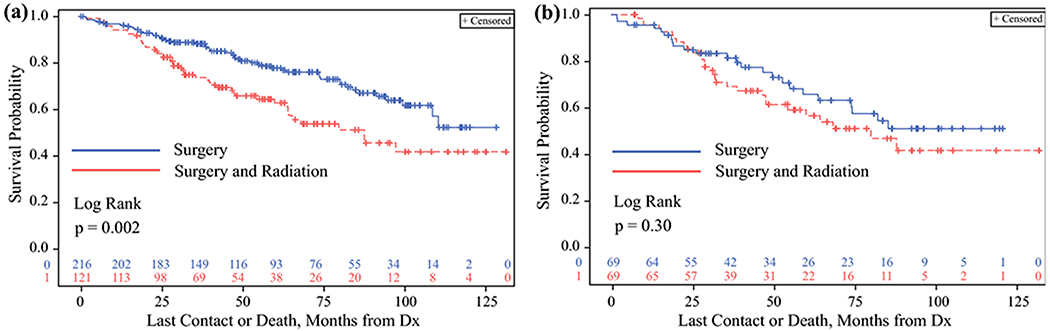
Kaplan-Meier analysis showing overall survival probability comparing patients with medullary thyroid cancer in the NCDB cohort who underwent surgery compared to those that underwent surgery and radiation among the subset of patients with positive margins after surgery (**A**) and among the subset of patients with positive margins in the propensity score matched cohort (**B**). Abbreviation: Dx – diagnosis.

Additionally, since a positive surgical margin is perhaps the most important clinical variable for which postoperative radiation therapy would be considered, we examined the subset of patients with positive surgical margins independently. Among the subjects with positive surgical margins (N= 337, N= 216 treated with S, N= 121 treated with SRT), overall survival remained significantly lower among the patients who received SRT (Figure 4A, Log rank p = 0.002). A propensity-matched analysis on this subset (N= 67 in each treatment group), is shown in Figure 4B. Although this analysis has modest statistical power, the same pattern was observed compared to the entire cohort, where similar or slightly improved survival in the SRT group was noted before 25 months, with a divergence showing lower survival in the SRT group after that timepoint.

### Analysis stratified by number of lymph nodes positive

In order to further control for potential treatment selection bias, the cohort was substratified by lymph node burden, a variable which could both influence treatment selection and outcome.^11^ Thus, a stratified analysis was carried out based on the number of lymph nodes positive for metastatic disease. The number of identified metastatic nodes were categorized based on quartile cutoffs: 1-3 lymph nodes positive, N=294; 4-7 lymph nodes positive, N=314; 8-16 lymph nodes positive, N=277; and >16 lymph nodes positive, N=230. Surgery with radiation was significantly associated with decreased overall survival in the three greatest quartiles, presented in Supplemental Figure 1 (1-3 lymph nodes positive, Log Rank p=0.09; 4-7 lymph nodes positive, Log Rank p=0.006; 8-16 lymph nodes positive, Log Rank p<0.0001; and >16 lymph nodes positive, Log Rank=0.0009).

## Discussion

MTC is a rare disease and therefore difficult to study. In the absence of distant metastasis, outcomes are highly dependent on the ability to achieve complete surgical eradication of disease. When a negative margin resection is not achievable, for example due to significant extrathyroidal extension or lymphatic burden, postoperative radiation may be recommended as a potential option to improve locoregional control, and perhaps survival.^6^ However, because prolonged survival is common among patients with MTC, even for those with advanced disease, benefits of postoperative radiation must be weighed against the long-term sequelae of radiation treatment. Proper evaluation of the benefits of postoperative radiation in this setting has been difficult due to a lack of data – the rarity of MTC has precluded the ability to examine the benefits of postoperative radiation in a prospective trial. Our current examination of data available in the NCDB suggests that postoperative radiation is associated with significantly lower overall survival among patients with advanced MTC.

With a paucity of data evaluating the risks and benefits of adjuvant radiation for MTC, recommendations rely on limited existing reports and expert opinion. The NCCN guidelines state that adjuvant locoregional radiation “may be considered for grossly incomplete resection when additional attempts at surgical resection have been ruled out”, and goes on to state that it is “rarely recommended”.^6^ Despite a limited role for postoperative radiation in this disease, approximately 19% of the patients in the NCDB cohort we evaluated with locoregionally advanced MTC received radiation. The ATA guidelines recommend locoregional adjuvant radiation for patients with “microscopic or macroscopic residual MTC, extrathyroidal extension, or extensive lymph node metastasis”, and for those “at risk of airway obstruction”^12^. As a basis for these recommendations, the NCCN guidelines notably cite the ATA guidelines, and the ATA guidelines reference small retrospective cohorts that report favorable locoregional control using postoperative radiation^7, 8, 13^. Similar to our study, a report by Martinez and colleagues studying SEER data did not find an association between radiation treatment and improved overall survival among patients with MTC and positive lymph node disease^10^. Though sparse, the collective data suggest that postoperative RT may improve locoregional control for some patients^7, 9, 10, 14^. However, the addition of radiation does not appear to improve overall survival. Our data imply that application of radiation is associated with decreased overall survival, however this deserves close scrutiny. Because all retrospective analyses such as this report suffer from selection biased and unmeasured confounders, one must consider a well-designed prospective study to evaluate the true risks and benefits of postoperative RT for this population.

The data supporting radiation for locoregionally advanced MTC were summarized in a recently published systematic review^14^. This review examined 23 publications that collectively described outcomes among a total of 1320 patients with MTC who were treated with surgery and postoperative radiation. Several of these papers described duplicate or overlapping cohorts, and all of the studies were either retrospective cohort studies or case series. Data did not clearly support an overall survival benefit with post-operative radiation. The major benefit of postoperative radiation appeared to be improvement in rates of locoregional relapse. The metanalysis carried out in this study suggested that postoperative radiation resulted in a 38% reduction in locoregional recurrence. This number was felt to be an under-estimate due to significant heterogeneity between studies and a tendency for patients with more advanced disease features to be selected for post-operative radiation^14^. In our analysis of the NCDB data, we could not evaluate locoregional recurrence as an outcome, as this information is not captured by the database^15^. Interestingly, however, the survival curves among our analyses do not seem to diverge until approximately the 2-year mark after diagnosis. In the propensity matched cohort, there appeared to be a slight survival advantage among the patients who received surgery with radiation until 2 years, at which point survival among the RT cohort decreases. It is possible that the benefits of locoregional treatment are realized at early time points, while perhaps the long-term toxicities of radiation take a greater toll at later time points. Alternatively, unmeasured confounders (such as persistent calcitonin levels, the presence or absence of targetable RET mutations) may be driving the decision to employ postoperative radiation. These limitations of the NCDB must be acknowledged, and only a carefully designed prospective study could definitively establish the proper setting for, and accurately characterize the benefits of, postoperative radiation for patients with advanced MTC.

We found radiation was associated with significantly lower survival even among patients with high lymph node burden. A recent NCDB analysis by Moses et al. emphasized the importance of lymph node disease burden and prognosis in MTC.^11^ This analysis elucidated that the five-year overall survival was approximately 95% for those without any nodal disease versus 75% in those with the highest nodal burden (>10 positive nodes). The authors found a statistically significant > 2-fold increase in death for patients with nodal disease within the multivariable analysis. In our own analysis, even after adjusting for lymph node burden both in a multivariable model and after propensity score matching, overall survival remained significantly (and markedly) lower in the group treated with postoperative radiation.

The long-term effects of radiation to the head and neck region, mostly studied among patients treated for head and neck squamous cell carcinoma, are well-described^16–18^. However, the common sequelae and prevalence of secondary effects are not directly translatable to patients who receive RT for MTC due to differences in dosing strategies and treatment planning for the two diseases. Commonly reported sequelae include xerostomia, neck fibrosis, dysphagia, esophageal stenosis, and carotid stenosis. Several studies have demonstrated that patients treated for head and neck cancer have increased risk of mortality from non-cancer competing risk factors^19, 20^, which include advanced age and comorbid conditions. Patients who have received neck radiation are known to be at increased risk of carotid artery atherosclerosis and ischemic stroke^21–24^. Because the long-term survival for patients with MTC are generally more favorable than for those with advanced head and neck squamous cancer, one could hypothesize that patients treated for MTC may bear a higher burden of long-term sequelae.

Our study of NCDB data has several limitations that warrant discussion. As mentioned above, because the database is composed of retrospective, population-based data, it is impossible to completely negate the selection-bias inherent to treatment allocation. We cannot measure or analyze all of the factors that may have influenced the decision to provide radiation treatment. For example, if patients were not offered radiation because calcitonin levels were undetectable (calcitonin level is not reported by NCDB), while others were offered radiation due to suspicion of residual disease based on serum biomarkers, then outcomes reflected in our analysis would be biased because the radiation cohort would inherently be more likely to develop relevant locoregional or distant disease. We attempted to control for known risk factors with both multivariable modeling and propensity score matching, but the potential for selection bias remains. Additionally, as mentioned above, the NCDB does not provide data to examine locoregional recurrence, so we could not examine whether radiation provided benefit using this outcome measure. There may exist coding or classification errors, unmeasured variables and/or specific treatment information that are inherent to any database study. Finally, the treatment for patients with advanced MTC is evolving rapidly, largely due to a growing understanding of the genomic and molecular characteristics of this disease^25^. Specifically, agents targeting activating RET-mutations have been highly effective^26^, enough so that a neodadjuvant approach for patients with RET-mutated MTC appears potentially effective^27^ and is under active investigation (NCT04759911). Since genomic information, such as RET-mutation status, is not yet captured by the NCDB, these factors are potential unmeasured confounders that will impact outcome in a contemporary cohort. Regardless of the limitations, our findings were striking, and suggest that proper indications for postoperative radiation with careful long-term outcome assessment for patients with advanced MTC deserve evaluation in a prospective manner.

Despite the limitations of using a retrospective dataset, our study represents the most contemporary and largest study comparing outcomes between patients who received S versus SRT for locoregionally advanced medullary thyroid carcinoma. Patients with advanced MTC in the NCDB cohort who received postoperative radiation experienced substantially lower overall survival. The indications for postoperative radiation and the long-term outcomes after this approach for patients with advanced MTC deserves close attention in prospective clinical trials.

## Supplementary Material

1775109_Sup_Fig_1 **Supplemental Figure 1.** Kaplan-Meier analysis showing overall survival probability comparing patients with medullary thyroid cancer in the NCDB cohort who underwent surgery compared to those that underwent surgery and radiation, stratified by subsets based on quartile of number of cervical lymph nodes positive. **(A)** Patients with 1 – 3 positive nodes, **(B)** 4 – 7 positive nodes, **(C)** 8-16 positive nodes, and **(D)** >16 positive nodes. Abbreviation: Dx – diagnosis.

## Figures and Tables

**Table 1.** Characteristics of the medullary thyroid cancer national cancer database dataset comparing the group that received surgery to the group that received surgery and radiation

Variable	Surgery Only		Surgery and Radiation		Total Cohort		p -value
	N	(%)	N	(%)	N	(%)	
Total	1112	81%	258	19%	1370	100%	
Year of Diagnosis							
2008 – 2011	324	29%	92	36%	416	30%	0.1
2011 – 2014	351	32%	69	27%	420	31%	
2014 – 2017	437	39%	97	38%	534	39%	
Age							
<40 years	227	20%	30	12%	257	19%	0.008 *
40-50 years	222	20%	69	27%	291	21%	
50-60 years	259	23%	58	22%	317	23%	
60-70 years	233	21%	61	24%	294	21%	
>70 years	171	15%	40	16%	211	15%	
Gender							
Male	557	50%	166	64%	723	53%	<0.001 *
Female	555	50%	92	36%	647	47%	
Race - Ethnicity							
non-Hispanic White	879	79%	206	80%	1085	79%	0.09
non-Hispanic Black	79	7%	26	10%	105	8%	
Hispanic	95	9%	20	8%	115	8%	
Other	59	5%	6	2%	65	5%	
Charlson-Deyo Comorbidity Score							
0	910	82%	212	82%	1122	82%	0.68
1	169	15%	36	14%	205	15%	
2 or 3	33	3%	10	4%	43	3%	
Insurance							
None	45	4%	14	6%	59	4%	0.43
Private	697	65%	149	60%	846	62%	
Medicaid	78	7%	21	8%	99	7%	
Medicare	255	24%	66	26%	321	23%	
Missing					45	3%	
Income Quartiles							
<$30,000	123	11%	26	10%	149	11%	0.22
$30, 000 - $34,999	130	12%	37	14%	167	12%	
$35,000 - $45,999	256	23%	73	28%	329	24%	
>$46,000	461	41%	93	36%	554	40%	
Missing	141	13%	29	11%	171	12%	
Surgical margin status							
Negative	840	76%	118	46%	958	70%	<0.0001 *
Positive	216	19%	121	47%	337	25%	
Missing	56	5%	19	7%	75	5%	
Extrathyroidal extension							
No	582	52%	58	22%	640	47%	<0.0001 *
Yes	302	27%	156	60%	458	33%	
Missing	228	21%	44	17%	272	20%	
AJCC T-stage							
T1	352	32%	30	12%	382	28%	<0.0001 *
T2	277	25%	31	12%	308	22%	
T3	346	31%	115	45%	461	34%	
T4	81	7%	68	26%	149	11%	
Tx	39	4%	9	3%	48	4%	
Missing	17	2%	5	2%	22	2%	
AJCC Stage Overall Stage							
III	442	40%	35	14%	477	35%	<0.0001 *
IV	670	60%	223	86%	893	65%	
Number of Lymph Nodes Positive							
1 to 3	303	27%	22	9%	325	24%	<0.0001 *
4 to 7	314	28%	51	20%	365	27%	
8 to 16	258	23%	75	29%	333	24%	
>16	215	19%	102	40%	317	23%	
Missing	22	2%	8	3%	30	2%	

* denotes below p<0.01 for statistical significance

abbreviations: AJCC - American Joint Commission on Cancer

**Table 2.** Multivariable cox regression for overall survival adjusting for multiple variables among the National Cancer Database cohort with medullary thyroid cancer.^A^

Variable	Hazard Ratio (HR)	95% HR C.I.	p-value
Treatment			
Surgery Alone	reference		
Surgery and Radiation	1.784	(1.313, 2.426)	*0.0002* *
Year of Diagnosis	1.005	(0.940, 1.075)	0.8804
Age			
<40 years	reference		
40-50 years	0.679	(0.374, 1.231)	0.2020
50-60 years	0.808	(0.478, 1.364)	0.4246
60-70 years	2.323	(1.510, 3.574)	0.0001 *
>70 years	2.639	(1.678, 4.149)	<0.0001 *
Gender			
Male	reference		
Female	0.716	(0.522, 0.980)	<*0.0372* *
Race-Ethnicity			
Non-Hispanic White	reference		
Non-Hispanic Black	1.056	(0.631, 1.767)	0.8343
Hispanic	0.253	(0.110, 0.580)	0.0012 *
Other	0.725	(0.293, 1.792)	0.4856
Charlson-Deyo Score			
0	reference		
1	0.963	(0.655, 1.418)	0.8497
2 or 3	2.49	(1.381, 4.493)	0.0024 *
T-Stage			
T1	reference		
T2	1.556	(0.892, 2.715)	0.1196
T3	1.922	(1.160, 3.186)	0.0113
T4	3.091	(1.773, 5.390)	<0.0001 *
Tx	4.425	(2.074, 9.441)	0.0001 *
Number of LN positive			
1 to 3	reference		
4 to 7	1.193	(0.733, 1.941)	0.4775
8 to 16	1.526	(0.934, 2.493)	0.0914
>16	2.139	(1.340, 3.417)	0.0015 *
Income			
<$30,000	reference		
$30, 000 - $34,999	0.783	(0.462, 1.327)	0.3628
$35,000 - $45,999	0.685	(0.434, 1.079)	0.1028
>$46,000	0.517	(0.332, 0.805)	0.0035 *
Surgical Margins			
Negative	reference		
Positive	1.678	(1.222, 2.304)	0.0014 *

* denotes below p<0.01 for statistical significance

abbreviations: HR - hazard ratio; CI – Confidence Interval

AJCC - American Joint Commission on Cancer

A Extra-thyroidal extension was not included in the final model because it was highly correlated with AJCC T-stage, and AJCC overall stage was not included because it was highly correlated with lymph node status and AJCC T-stage.

## Synopsis

A contemporary analysis using the National Cancer Database examining post-surgical radiation treatment among patients with advanced medullary thyroid carcinoma (MTC) did not demonstrate increased survival. Prospective studies are necessary to establish clear guidelines for adjuvant radiation in this setting.
