# Supplementary figures and images for: Evaluation of Survival and Post-operative Radiation Among Patients with Advanced Medullary Thyroid Carcinoma: An Analysis of the National Cancer Database
Source: Ann Surg Oncol. Author manuscript; Available in PMC 2023 Apr 1. (PMC8940671; doi:10.1245/s10434-021-11158-9)

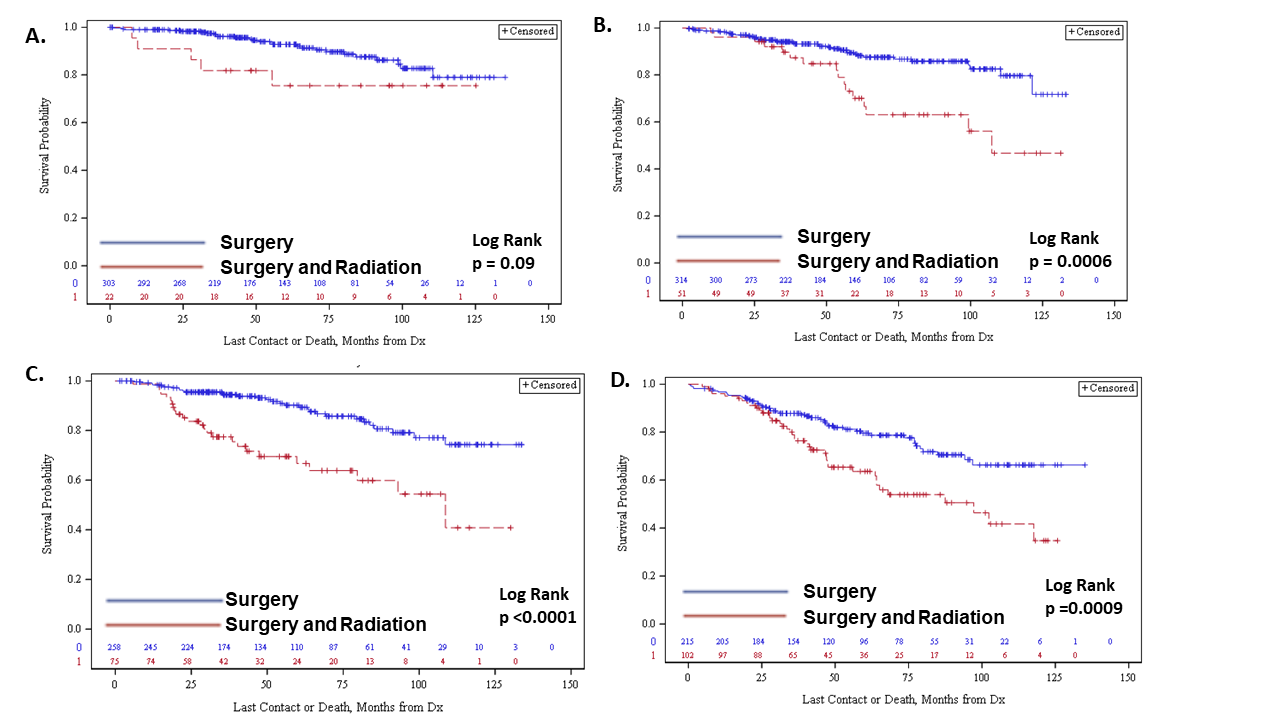

Supplement: 1775109_Sup_Fig_1 — Supplemental Figure 1. Kaplan-Meier analysis showing overall survival probability comparing patients with medullary thyroid cancer in the NCDB cohort who underwent surgery compared to those that underwent surgery and radiation, stratified by subsets based on quartile of number of cervical lymph nodes positive. (A) Patients with 1 – 3 positive nodes, (B) 4 – 7 positive nodes, (C) 8-16 positive nodes, and (D) >16 positive nodes. Abbreviation: Dx – diagnosis. [file NIHMS1775109-supplement-1775109_Sup_Fig_1.tif]
